# *Para*-Aminobenzohydrazide Derivatives as Fatty Acid Amide Hydrolase Inhibitors: Design, Synthesis and Biological Evaluation

**DOI:** 10.22037/ijpr.2020.113899.14551

**Published:** 2020

**Authors:** Anna Sedaghat, Elham Rezaee, Omid Hosseini, Sayyed Abbas Tabatabai

**Affiliations:** a *Department of Pharmaceutical Chemistry, School of Pharmacy, Shahid Beheshti University of Medical Sciences, Tehran, Iran. *; b *Centra Research Labretories, Shahid Beheshti University of Medical Sciences, Tehran, Iran.*

**Keywords:** 4-Aminobenzohydrazide, Docking, Fatty acid amide hydrolase, Inhibitor, Synthesis

## Abstract

The endocannabinoid system plays an important neuromodulatory role in the periphery and central nervous system, which can regulate several physiological processes. The inhibition of enzymatic activities responsible for hydrolysis anandamide and other endogenous fatty acid amides, enhances cannabinoid receptors activity indirectly that may prove to be useful drugs for the treatment of range of ailments including pain, anxiety, and other central nervous system disorders. In this study, we designed, synthesized, and evaluated novel fatty acid amide hydrolase (FAAH) inhibitors based on 4-aminobenzohydrazide derivatives. Most of the synthesized compounds exhibited a proper affinity for the catalytic triad of FAAH in docking studies and had a considerable *in-vitro* FAAH inhibitory activity in comparison with JZL-195, a potent inhibitor of FAAH. Compound 2-(2-(4-(2-carboxybenzamido) benzoyl) hydrazine-1-carbonyl) benzoic acid, 12, was found to be the most potent inhibitor with IC_50_ value of 1.62 nM targeting FAAH enzyme.

## Introduction

**Figure 1:**
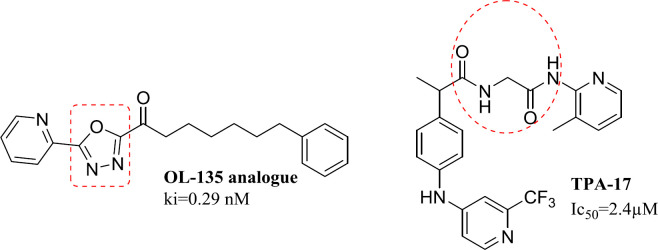
Chemical structures of the OL-135 analogue and Ibuprofen amide as known FAAH inhibitors

Fatty acid amides hydrolase (FAAH) is a member of the serine hydrolase enzyme family responsible for breaking down fatty amides, the body’s first line of defense against pain (1, 2). Anandamide is a naturally occurring brain constituent with agonist activity against cannabinoid receptors (3, 4). A decrease in FAAH activity increases the levels of various endogenous endocannabinoids. This strategy is particularly useful to modulate cannabinoid receptors and produce therapeutic effects with minimal risk of adverse cannabis-like side effects (5)or endocannabinoids. FAAH inhibitors have shown analgesic and antiinflammatory activity in animal models, and some have been tested in phase 1 and 2 studies. In a phase 1 study, BIA 10-2474, an orally administered reversible FAAH inhibitor, was given to healthy volunteers to assess safety. METHODS Single doses (0.25 to 100 mg. FAAH inhibitors have shown pharmacological properties in reducing the pain, inﬂammation, anxiety, and regulation of mood as well (6, 7)through the hydrolysis of the endocannabinoids anandamide and in some tissues 2-arachidonoylglycerol. FAAH inhibition represents a promising strategy to activate the cannabinoid system, since it does not result in the psychotropic and peripheral side effects characterizing the agonists of the cannabinoid receptors. Here we present the discovery of a novel class of profen derivatives, the N-(heteroaryl. The main types of FAAH inhibitors, including reversible and irreversible inhibitors, exert their effects by interacting with Ser_217_-Lys_142_ - Ser_241_ in the catalytic triad (8). It is previously reported that TPA-17 displays mild FAAH inhibitory activity and conversion of the carboxylic group into amide increases FAAH inhibitory activity. (Figure 1) Furthermore, most potent reversible inhibitors reported to date bearing an oxadiazole ring as seen in OL-135 analogue (6, 8 and 9) (Figure 1).

**Figure 2:**
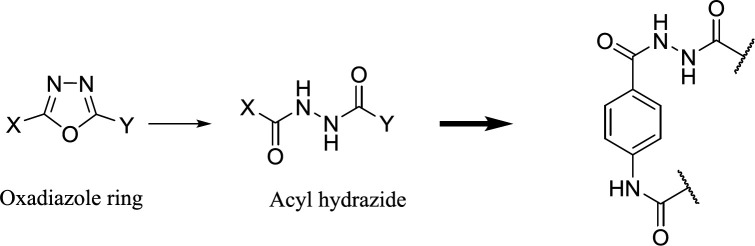
Open the form of oxadiazole ring in chemical structures of the designed compounds as FAAH inhibitor

On the other hand, one of the effective methods in drug design is the ring-opening technique, (10)ring systems, and frameworks in drugs listed in the FDA Orange Book to understand the frequency, timelines, molecular property space, and the application of these rings in different therapeutic areas and target classes. This analysis shows that there are only 351 ring systems and 1197 frameworks in drugs that came onto the market before 2013. Furthermore, on average six new ring systems enter drug space each year and approximately 28% of new drugs contain a new ring system. Moreover, it is very unusual for a drug to contain more than one new ring system and the majority of the most frequently used ring systems (83% and we can imagine the oxadiazole ring in its open acyl hydrazide form (11). In practice, we used this principle to design novel derivatives of 4-aminobenzohydrazide as FAAH inhibitors. (Figure 2)

The docking study was also performed to better understand the spatial orientation of the synthesized compounds in the FAAH active site and finally biological evaluation was applied to measure the inhibitory activity of the compounds. 

## Experimental

*Molecular Modeling Studies*

Spatial interaction in FAAH active site was simulated using AutoDock 4.0 software (Lamarckian genetic algorithm) to predict the interaction of the synthesized compounds. The high-resolution crystal structure of FAAH with OL-135 as a ligand was retrieved from RCSB Protein Data Bank (PDB code: 3PPM). The structure of the enzyme was assumed to be rigid, and the ligands were considered flexible and polar hydrogens and Kollman united atom partial charges were added. The HyperChem 8 software was used for energy minimization of each structure under MM + method and AutoDockTools 4.0 version 1.5.6rc3 to render pdbqt format. A docking grid box was built with 40, 40, and 40 points in the catalytic site of protein and the number of generations and maximum number of energy evaluations was set to 100 and 2,700,000, respectively. The docking results were clustered with RMSD = 0.5 Å and evaluated by Pymol software (12).

*Chemistry*

All chemicals and solvents were purchased from Merck Company and used without further purification. TLC was performed on commercially available Merck plates (silica gel 60 F254, 0.25 mm). Electrothermal 9100 apparatus was used to measure uncorrected melting points. Infrared spectra were obtained on a Perkin Elmer 843 spectrometer. A Bruker FT-400 MHz instrument (Bruker Biosciences, USA) was used to obtain ^1^HNMR and ^13^CNMR spectra in DMSO d_6_, using TMS as an internal standard. Chemical shifts were stated as ppm against TMS as the internal standard. Coupling constant (J) increments are estimated in hertz (Hz) and spin multiples are shown as “s” for “singlet”, “d” for “doublet”, “t” for “triplet”, “q” for the “quartet”, “m” for “multiplet”, and “br” for “broad” signal. LC Mass spectra and elemental analysis were achieved by HPLC Agilent system and Costech elemental analyzer respectively. 

*Ethyl 4-aminobenzoate (* ***2*** *)*

To 4-aminobenzoic acid 1 (13.7 g, 0.1 mol) in ethanol (100 mL), 10 mL of concentrated sulfuric acid was added and the mixture was refluxed for 12 h. The resulting mixture was concentrated under vacuum and then poured into 200 mL of water. The resulting solution was slowly neutralized to pH 8 using solution of potassium carbonate. The final solid was separated and slowly washed with water (100) and finally dried in a vacuum (13). Yield 90%, m.p: 90-92 °C, IR (KBr) ν (cm-1) 3100-3500 (NH2), 1630 (C = O), LC-MS (ESI) m/z 166 (M + H).

*General procedure for the synthesis of the esters 3a-3b*

To the suspension of ethyl 4-aminobenzoate **2** (0.66 g, 4 mmol) in DCM (10 m, 0 °C), DIPEA (0.59 g, 0.8 mL) was added. Then 4.4 mmol of benzoyl halide was added and stirred for 12 h at room temperature. DCM (50 mL) was added to the mixture to obtain a clear solution. The resulting organic phase was washed twice with HCl (50 mL, 2 M). The collected organic phase was dried over anhydrous sodium sulfate and evaporated under vacuum to give a crystalline product (14). 

*Ethyl 4-benzamidobenzoate (3a)*

Yield 92%, m.p: 149-150 °C, IR (KBr) ν (cm^-1^) 3300 (NH), 1700 (C = O), 1650 (C = O), 1200-1400 (Ar), LC-MS (ESI) m/z 268 (M-H).

*Ethyl 4-(4-chlorobenzamido) benzoate (3b)*

Yield 95%, m.p: 173-75 °C, IR (KBr) ν (cm^-1^) 3300 (NH), 1700, 1650 (C = O), 1200-1400 (Ar), LC-MS (ESI) m/z 302 (M-H).

*General procedure for the synthesis of the hydrazide 4a-4b, 6*

Benzoate ethyl ester **2 **or** 3a-3b** (5 mmol) and hydrazine hydrate (20 mmol) were melted (110 °C, 90 min) in a closed container. Recrystallization from EtOH yielded intermediate hydrazide (15). 

*N-(4-(hydrazinecarbonyl) phenyl) benzamide (* ***4a*** *)*

Yield 65%, m.p: 321-324 °C, IR (KBr) ν (cm^-1^) 3300-3200 (NH, NH_2_), 1670, 1650 (C = O), LC-MS (ESI) m/z 256 (M+H).

*4-chloro-N-(4-(hydrazinecarbonyl) phenyl) benzamide (* ***4b*** *)*

Yield 55%, m.p: 335-340 °C, IR (KBr) ν (cm^-1^) 3300-3200 (NH, NH_2_), 1670, 1650 (C = O), LC-MS (ESI) m/z 290 (M+H).

*4-aminobenzohydrazide (* ***6*** *)*

Yield 85%, m.p: 225-228 °C, IR (KBr) ν (cm^-1^) 3400-3100 (NH, NH_2_), 1670 (C = O), LC-MS (ESI) m/z 152 (M+H).

*General procedure for the synthesis of the compounds* ***5a, 5b, 7***

The intermediate hydrazide compounds **6** or **4a-4b** (1 mmol) in cold methanol (3-5 mL, 5 °C) and in the presence of acetic acid (0.24 g, 4 mmol) were stirred at room temperature for 15 min. Potassium cyanate (0.324 g, 4 mmol) was added within 15min. Then put the lid and continue to stir for 24 h at room temperature. Water (2 mL) was added to the reaction vessel and the resulting white precipitate was separated and washed with warm water (10 mL, 50 °C) (14). 

*2-(4-benzamidobenzoyl) hydrazine-1-carboxamide (* ***5a*** *)*

Yield: 56%, m.p: 283-285 °C, IR (KBr) ν (cm^-1^) 3350-3000 (NH, NH_2_), 1750-1650 (C = O), 1200-1400 (Ar). ^1^H NMR (400 MHz, DMSO) δ*ppm *10.51 (s, 1H, NH), 10.09 (s, 1H, NH), 8.01–7.95 (m, 2H, H_2_, H_6_-phenylene), 7.94–7.84 (m, 4H, H-benzoyl, NH), 7.66–7.51 (m, 4H, phenyl, H_3_, H_5_-phenylene), 6.05 (s, 2H, NH_2_).^13^C NMR (101 MHz, DMSO) δ*ppm* 166.35, 166.26, 142.64, 135.16, 132.28, 128.92, 128.79, 128.23, 128.11, 119.94, 119.82. LC-MS (ESI) m/z 299 (M + H). Anal.Calcd for C_15_H_14_N_4_O_3_: C, 60.40; H, 4.73; N, 18.78. Found: C, 60.34; H, 4.70; N, 18.92. 

*2-(4-(4-chlorobenzamido) benzoyl) hydrazine-1-carboxamide (* ***5b*** *)*

Yield: 59%, m.p: 325-327 °C, IR (KBr) ν (cm^-1^) 3350-3000 (NH, NH_2_), 1750-1650 (C = O), 1200-1400 (Ar). ^1^H NMR (400 MHz, DMSO) δ*ppm *10.58 (s, 1H, NH), 10.08 (s, 1H, NH), 8.05–7.95 (d, *J* = 8.4 Hz, 2H, H_2_, H_6_-phenylene), 7.93–7.82 (m, 5H, -phenylene, NH), 7.65–7.57 (d, *J* = 8.4 Hz, 2H, H_3_, H_5_-phenylene), 6.05 (s, 2H, NH_2_ ).^13^C NMR (101 MHz, DMSO) δ*ppm* 166.45, 165.42, 142.37, 137.18, 133.73, 130.17, 129.03, 128.81, 128.31, 128.14, 120.17, 120.01. LC-MS (ESI) m/z 333 (M + H), 355 (M + Na). Anal.Calcd for C_15_H_13_ClN_4_O_3_: C, 54.15; H, 3.94; N, 16.84. Found: C, 54.22; H, 3.91; N, 16.80.

*2-(4-ureidobenzoyl) hydrazine-1-carboxamide (* ***7*** *)*

Yield: 45%, m.p: 226-228 °C (dc), IR (KBr) ν (cm^-1^) 3500-3000(NH, NH_2_), 1700-1600 (C = O), 1200-1400(Ar). ^1^H NMR (400 MHz, DMSO) δ*ppm *9.95 (s, 1H, NH), 8.84 (s, 1H, NH), 7.79 (d, *J* = 8.4 Hz, 2H, H_2_, H_6_-phenylene), 7.72 (s, 1H, NH-urea), 7.47 (d, *J* = 8.4 Hz, 2H, H_3_, H_5_-phenylene), 6.02 (s, 2H, NH_2_), 5.69 (s, 2H, NH_2_). ^13^C NMR (101 MHz, DMSO) δ*ppm* 166.83, 156.22, 152.48, 144.18, 129.61, 125.42, 119.64. LC-MS (ESI) m/z 236 (M-H). Anal.Calcd for C_9_H_11_N_5_O_3_: C, 45.57; H, 4.67; N, 29.52. Found: C, 45.48; H, 4.70; N, 29.56.

*General procedure for the synthesis of the compounds* ***8, 9***

Method a: *Para*-aminobenzohydrazide **6** (0.151g, 1 mmol) was refluxed with succinic anhydride or phthalic anhydride (2.02 mmol) in pyridine (5 mL) for 72 h. Cold water (50 mL) was added and the reaction was acidified with concentrated HCl. The resulting precipitate was filtered off and washed with water. 

Method b: an effective alternative method was to melt the reactant mixture at 140 °C for 4 h. The resulting raw product was recrystallized from ethyl acetate and, if necessary, purified by a short column of silica gel and mobile phase of Hexane: chloroform (20: 80). 

*N, 4-bis (1,3-dioxoisoindolin-2-yl) benzamide (* ***8*** *)*

Yield: 75%, m.p: 316-318 °C, IR (KBr) ν (cm^-1^) 3350 (NH), 1750-1650 (C = O), 1400-1100 (Ar). ^1^H NMR (400 MHz, DMSO) δ*ppm *11.47 (s, 1H, NH), 8.12 (d, *J* = 8 Hz, 2H, H_2_, H_6_-phenylene), 8.08 – 7.08 (m, 8H, 2 phthalimide), 7.68 (d, *J* = 8 Hz, 2H, H_3_, H_5_-phenylene). ^13^C NMR (101 MHz, DMSO) δ*ppm* 167.14, 166.27, 165.81, 136.62, 135.96, 135.80, 135.36, 130.38, 130.09, 130.02, 129.96, 127.75, 124.42, 124.23, 124.07. LC-MS (ESI) m/z 410 (M-H). Anal.Calcd for C_23_H_13_N_3_O_5_: C, 67.15; H, 3.19; N, 10.21. Found: C, 67.11; H, 3.21; N, 10.25.

*N, 4-bis (2,5-dioxopyrrolidin-1-yl) benzamide* *(****9*** *)*

Yield: 48%, m.p: 273-275 °C, IR (KBr) ν (cm^-1^) 3350 (NH), 1750-1650 (C = O), 1400-1100 (Ar). ^1^H NMR (400 MHz, DMSO) δ*ppm *9.56 (s, 1H, NH), 7.88 (d, *J* = 8.8 Hz, 2H, H_2_, H_6_-phenylene), 7.14 (d, *J* = 8.8 Hz, 2H, H_3_, H_5_-phenylene), 2.42 (m, 8H, 2 succinimide). ^13^C NMR (101 MHz, DMSO) δ*ppm* 174.05, 167.33, 143.40, 131.42, 130.16, 125.39, 123.85, 130.29, 29.24. LC-MS (ESI) m/z 314 (M-H). Anal.Calcd for C_15_H_13_N_3_O_5_: C, 57.14; H, 4.16; N, 13.33. Found: C, 57.20; H, 4.12; N, 13.30.

*General procedure for the synthesis of the compounds* ***10a-10f***

*Para*-aminobenzohydrazide **6** (0.151 g, 1 mmol) with the proper acyl halides (2.02 mmol) was stirred in pyridine (5 mL) for 24 h. The reaction mixture was poured into 30 mL of distilled water, stirred for 5 min and acidified with concentrated HCl. The final product was recrystallized from EtOH. 

*N-(4-(2-benzoylhydrazine-1-carbonyl) phenyl) benzamide (* ***10a*** *)*

Yield: 65%, m.p: 265-267 °C, IR (KBr) ν (cm^-1^) 3350-3100 (NH), 1750-1650 (C = O), 1200-1400 (Ar) ^1^H NMR (400 MHz, DMSO) δ*ppm *10.56–10.44 (m, 3H, 3 NH), 8.02–7.91 (m, 8H, H_2_, H_6_-benzoyl, H-phenylene), 7.67–7.47 (m, 6H, H_3_, H_4_, H_5_-benzoyl).^13^C NMR (101 MHz, DMSO) δ*ppm* 166.41, 166.37, 165.82, 142.89, 135.16, 133.09, 132.32, 128.99, 128.93, 128.75, 128.26, 127.94, 127.81, 120.02 LC-MS (ESI) m/z 358 (M-H). Anal.Calcd for C_21_H_17_N_3_O_3_: C, 70.18; H, 4.77; N, 11.69. Found: C, 70.14; H, 4.79; N, 11.65. 

*3-chloro-N-(4-(2-(3-chlorobenzoyl) hydrazine-1-carbonyl) phenyl) benzamide *(***10b****)*

Yield: 69%, m.p: 273-275 °C, IR (KBr) ν (cm^-1^) 3300-3100 (NH), 1750-1650 (C = O), 1200-1400 (Ar). ^1^H NMR(400 MHz, DMSO) δ*ppm *10.63 (s, 2H, 2NH), 10.53 (s, 1H, NH), 8.04 (d, *J* = 1.9 Hz, 1H, H_2_-benzoyl), 7.99–7.87 (m, 7H, -benzoyl, -phenylene), 7.70 (dd, *J* = 8.2, 2.0 Hz, 2H, H_2_, H_6_-phenylene), 7.59 (t, *J* = 7.9, Hz, 2H, -benzoyl).^13^C NMR (101 MHz, DMSO) δ*ppm* 165.71, 164.99, 164.91, 142.42, 137.09, 134.99, 133.85, 133.73, 132.24, 132.13, 131.11, 130.96, 128.79, 128.00, 127.91, 127.73, 127.10, 126.67, 120.13 LC-MS (ESI) m/z 426 (M-H). Anal.Calcd for C_21_H_15_Cl_2_N_3_O_3_: C, 58.90; H, 3.53; N, 9.81. Found: C, 58.84; H, 3.55; N, 9.85. 

*4-chloro-N-(4-(2-(4-chlorobenzoyl) hydrazine-1-carbonyl) phenyl) benzamide *(***10c****) *

Yield: 70%, m.p: 315-318 °C, IR (KBr) ν (cm^-1^) 3300-3100 (NH), 1750-1650 (C = O), 1200-1400 (Ar). ^1^H NMR (400 MHz, DMSO) δ*ppm *10.60 (d, *J* = 4.4 Hz, 2H, 2NH), 10.49 (s, 1H, NH), 8.02 (d, *J* = 8.4 Hz, 2H, H_3_, H_5_-phenylene), 7.96 (t, *J* = 5.9 Hz, 6H, H_3_, H_5_-phenylene, H_2_, H_6_-benzoyl), 7.63 (t, *J* = 8.0 Hz, 4H, H_3_, H_5_-benzoyl) ^13^C NMR (101 MHz, DMSO) δ*ppm* 165.78, 165.37, 165.27, 142.72, 137.21, 137.17, 133.81, 131.79, 130.24, 129.86, 129.15, 129.01, 128.78, 127.87, 120.10 LC-MS (ESI) m/z 426 (M-H). Anal.Calcd for C_21_H_15_Cl_2_N_3_O_3_: C, 58.90; H, 3.53; N, 9.81. Found: C, 58.96; H, 3.50; N, 9.78.

*4-methoxy-N-(4-(2-(4-methoxybenzoyl) hydrazine-1-carbonyl) phenyl) benzamide (* ***10d*** *)*

Yield: 75%, m.p: 264-266 °C, IR (KBr) ν (cm^-1^) 3300-3100 (NH), 1700-1650 (C = O), 1200-1400 (Ar). ^1^H NMR(400 MHz, DMSO) δ*ppm *10.36 (s, 3H, 3NH), 7.99 (d, *J* = 8.8 Hz, 2H, H_2_, H_6_-benzoyl), 7.92 (d, *J* = 5.5 Hz, 6H, H_2_, H_3_, H_5_, H_6_-phenylene, H_2_, H_6_-benzoyl), 7.07 (m, 4H, H_3_, H_5_-benzoyl), 3.85 (s, 3H, OMe), 3.84 (s, 3H, OMe) ^13^C NMR (101 MHz, DMSO) δ*ppm* 165.89, 165.69, 162.58, 162.47, 143.04, 130.24, 128.68, 127.62, 127.10, 125.22, 119.93, 114.19, 114.15, 55.94, 55.87 LC-MS (ESI) m/z 418 (M-H). Anal.Calcd for C_23_H_21_N_3_O_5_: C, 65.86; H, 5.05; N, 10.02. Found: C, 65.94; H, 5.02; N, 10.00. 

*4-fluoro-N-(4-(2-(4-fluorobenzoyl) hydrazine-1-carbonyl) phenyl) benzamide* *(****10e****)*

Yield: 63%, m.p: 322-325 °C, IR (KBr) ν (cm^-1^) 3700-3100 (NH), 1700-1650 (C = O), 1200-1400 (Ar). ^1^H NMR (400 MHz, DMSO) δ*ppm *10.55 (s, 2H, 2NH), 10.51–10.46 (m, 1H, NH), 8.05 (dd, *J* = 8.4, 5.4 Hz, 4H, H_2_, H_6_-benzoyl), 7.98–7.89 (m, 4H, H_2_, H_3_, H_5_, H_6_-phenylene), 7.39 (d, *J* = 8.7 Hz, 4H, H_3_, H_5_-benzoyl) ^13^C NMR (101 MHz, DMSO) δ*ppm* 165.93, 165.81, 165.32, 165.26, 163.45, 142.80, 131.55, 131.09, 131.00, 130.69, 130.60, 128.75, 127.81, 120.06, 116.13, 116.01, 115.92, 115.79 LC-MS (ESI) m/z 394 (M-H). Anal.Calcd for C_21_H_15_F_2_N_3_O_3_: C, 63.80; H, 3.82; N, 10.63. Found: C, 63.74; H, 3.86; N, 10.61. 

*4-nitro-N-(4-(2-(4-nitrobenzoyl) hydrazine-1-carbonyl) phenyl) benzamide (* ***10f*** *)*

Yield: 71%, m.p: 287-289 °C, IR (KBr) ν (cm^-1^) 3300-3100 (NH), 1700-1650 (C = O), 1350, 1550 (NO), 1200-1400 (Ar) ^1^H NMR (400 MHz, DMSO) δ*ppm *10.86 (s, 1H, NH), 10.72 (s, 2H, 2NH), 8.40 (m, 4H, H_3_, H_5_-benzoyl), 8.26–8.15 (m, 4H, H_2_, H_6_-benzoyl), 8.02–7.85 (m, 4H, H_2_, H_3_, H_5_, H_6_-phenylene) ^13^C NMR (101 MHz, DMSO) δ*ppm* 165.53, 164.76, 149.83, 149.75, 142.45, 140.77, 138.85, 129.83, 129.44, 128.84, 128.17, 124.25, 124.08, 120.25 LC-MS (ESI) m/z 448 (M-H). Anal.Calcd for C_21_H_15_N_5_O_7_: C, 56.13; H, 3.36; N, 15.58. Found: C, 56.09; H, 3.39; N, 15.55. 

*General procedure for the synthesis of the compounds* ***11-12***

*Para*-aminobenzohydrazide **6** (0.151 g, 1 mmol) with the desired anhydrides (2.02 mmol) was stirred in warm ethyl acetate (50 °C) for 30 min. Then, stirring was continued for 24 h at room temperature. The reaction mixture was poured into 30 mL of distilled water, stirred for 30min. and acidified with concentrated HCl. The final product was recrystallized from EtOAc.

*4-(2-(4-(3-carboxypropanamido) benzoyl) hydrazineyl)-4-oxobutanoic acid (* ***11*** *)*

Yield: 55%, m.p: 185-187 °C, 194 dc, IR (KBr) ν (cm^-1^) 3200-2800 (NH, COOH), 2750-2600 (-CH_2_-CH_2_), 1700 (C = O), 1200-1400 (Ar). ^1^H NMR(400 MHz, DMSO) δ*ppm *11.66 (br s, 2H, 2 COOH), 10.32-10.23 (d, 3H, 3NH), 7.80 (d, *J* = 8.5 Hz, 2H, H_2_, H_6_-phenylene), 7.67 (d, *J* = 8.5 Hz, 2H, H_3_, H_5_-phenylene), 2.60 (d, *J* = 6.3 Hz, 2H,-CH_2_-), 2.55 (d, *J* = 6.1 Hz, 2H, -CH_2_-), 1.99-1.94 (d, 4H,-CH_2_-) ^13^C NMR (101 MHz, DMSO) δ*ppm* 174.30, 171.00, 163.11, 160.17, 142.45, 129.03, 128.56, 118.45, 31.56, 29.12, 25.56, 18.45 LC-MS (ESI) m/z 350 (M-H). Anal.Calcd for C_15_H_17_N_3_O_7_: C, 51.28; H, 4.88; N, 11.96. Found: C, 51.20; H, 4.91; N, 12.04. 

*2-(2-(4-(2-carboxybenzamido) benzoyl) hydrazine-1-carbonyl) benzoic acid *(***12****)*

Yield: 85%, m.p: 186 °C, 197 dc, IR (KBr) ν (cm^-1^) 3300-2600 (NH, COOH), 1700 (C = O), 1200-1400 (Ar). ^1^H NMR(400 MHz, DMSO) δ*ppm *13.04 (br s, 2H, 2 COOH), 10.60 (s, 1H, NH), 10.325 (s, 2H, 2NH), 7.90 (d, *J* = 7.6 Hz, 2H, H, H’ *ortho*-phthalic), 7.85–7.70 (m, 5H, phthalic, H_2_, H_6_-phenylene), 7.69–7.54 (m, 5H, phthalic, H_3_, H_5_-phenylene) ^13^C NMR (101 MHz, DMSO) δ*ppm* 168.19, 167.83, 163.12, 160.32, 142.72, 139.14, 132.30, 130.29, 130.08, 130.02, 128.94, 128.26, 119.04 LC-MS (ESI) m/z 446 (M-H). Anal.Calcd for C_23_H_17_N_3_O_7_: C, 61.75; H, 3.83; N, 9.39. Found: C, 61.70; H, 3.85; N, 9.42. 

*N-(4-(2-(phenylsulfonyl) hydrazine-1-carbonyl) phenyl) benzenesulfonamide (* 13 *)*

Compound **13** was obtained following the procedure described for the synthesis of **10a-10f**. 

Yield: 51%, m.p: 239-241 °C, IR (KBr) ν (cm^-1^) 3700-3300 (NH), 1700-1650 (C = O), 1200-1400 (Ar), 1100-1200 (SO_2_) ^1^H NMR (400 MHz, DMSO) δ*ppm *11.40 (s, 1H, NH), 10.88 (s, 2H, 2NH), 7.97–7.50 (m, 14H, H_2_, H_3_, H_5_, H_6_-phenylene, benzenesulfonyl) ^13^C NMR (101 MHz, DMSO) δ*ppm* 166.03, 142.33, 139.68, 138.63, 135.10, 133.76, 129.94, 129.67, 129.24, 128.97, 128.05, 127.14, 126.29, 118.65 LC-MS (ESI) m/z 430 (M-H). Anal.Calcd for C_19_H_17_N_3_O_5_S_2_: C, 52.89; H, 3.97; N, 9.74. Found: C, 52.80; H, 3.94; N, 9.78. 

*In-vitro biological activity*

The inhibitory activity of the final products was evaluated against fatty acid amide hydrolase with Cayman plate-based fluorometric FAAH assay kit (item number 10005196) using an excitation wavelength of 340 nm and an emission wavelength of 450 nm. The enzyme was incubated with inhibitors for 5 min in 0.125 mM Tris/HCl buffer (pH 9.0) at 37 °C. AMC-arachidonoyl amide and JZL-195 were used as substrate and positive control respectively. All test samples were dissolved in DMSO. 

## Results and Discussion

*Molecular modeling studies*

**Figure 3:**
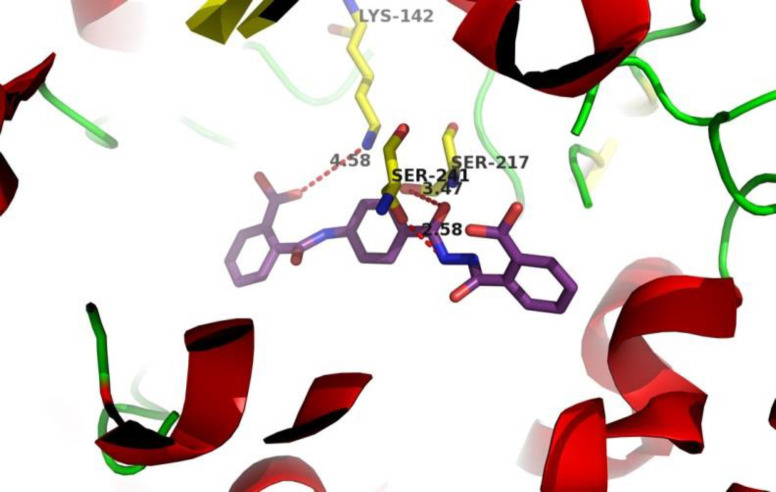
Interaction of compound **12** (purple) in the catalytic triad of FAAH

The interaction of final products in the FAAH catalytic site was simulated using docking to obtain Gibbs free energy results. The orientation of the most potent inhibitor **12** in the active site of FAAH was evaluated as shown in Figure 3. Interestingly, the carboxylic acid group in the compound **12 **is very close to Lys142, which increases the chances of ionic interaction and it is the potential reason for high inhibitory activity. Additionally, acyl hydrazide moiety in the compound **12 **has a suitable distance from the two critical amino acids: Ser217 and Ser241 for effective hydrogen bonding to potentiate its potency. 

*Chemistry*

**Scheme 1:**
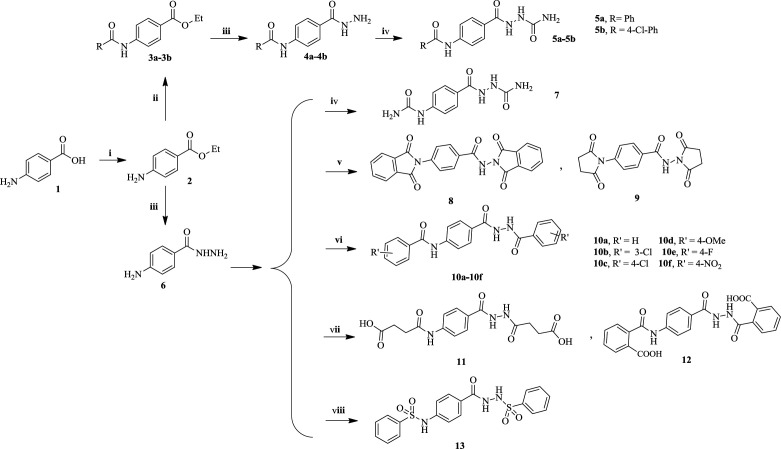
Synthesis of the designed compounds. Reagents and conditions: (i) EtOH, H_2_SO_4_, reflux, 12 h. (ii) Benzoyl halide, DIPEA, DCM, 12h. (iii) N_2_H_4_, 110 °C, 90 min, closed vessel. (iv) Portionwise addition of KCNO, MeOH, H^+^, r.t., 24 h. (v) Phthalic anhydride or succinic anhydride, pyridine, reflux, 72 h or dry melting at 150 °C, 2 h. (vi) Substituted benzoyl chloride or suitable anhydride, pyridine or EtOAC, r.t., 24 h. (vii) Succinic or phthalic anhydride, EtOAC, 50 °C, 30 min, then r.t., 24 h. (viii) Benzenesulfonyl chloride, pyridine, r.t., 24 h

The designed compounds were synthesized according to Scheme 1. Initially, *para*-aminobenzoic acid **1** (PABA) was reacted with ethanol under Fischer esterification to obtain benzocaine **2**, which was then converted to intermediate esters **3a-3b** through the acylation of NH_2_ group in an alkaline environment. Then, compounds **7** and **5a**-**5b** were obtained by reaction of esters **2** and **3a**-**3b** with N2H4 followed by treatment with potassium cyanate in an acidic solution in pressure vessel at temperatures above 110 °C. Finally, the reaction of 4-aminobenzohydrazide **6** with succinic or phthalic anhydride at high temperatures produces compounds **8**-**9**, while the reaction at a mild temperature in ethyl acetate affords compounds **11**-**12** bearing carboxylic acid moiety. Finally, treatment of 4-aminobenzohydrazide **6** with various benzoyl halides or sulfonyl halide in pyridine gave final products **10a-10f** and **13**.

*In-vitro biological activity*

All of the designed compounds showed good inhibitory activity against FAAH at concentrations of 100 nM. The IC_50_ of the compounds with inhibitory activity above 70% and JZL-195, reference compound, were investigated at concentrations of 0.1, 5, 10, 20, 50 and 100 nM. The results are summarized in Table 1 Evaluation of the **5a, 5b **and** 7** derivatives have well illustrated the effect of phenyl group on inhibitory activity, which is probably directly related to lipophilicity and π-π interactions in lipophilic envelope. Comparing the compounds **10a-10f, 11-12** with compounds **8-9** shows the importance of structural flexibility as well as possible π-π interaction which describes the difference in inhibitory effect between the two compounds **10a** and **8**. On the other hand, the presence of carboxylic acid functional group on the phenyl ring increases the inhibitory effect significantly. In contrast, the sulfonamide group does not play an effective role in inhibitory activity. These data represent a very good correlation with Gibbs free energy of the docking study. 

**Table 1:** FAAH Inhibitory activity of the 4-aminobenzohydrazide derivatives

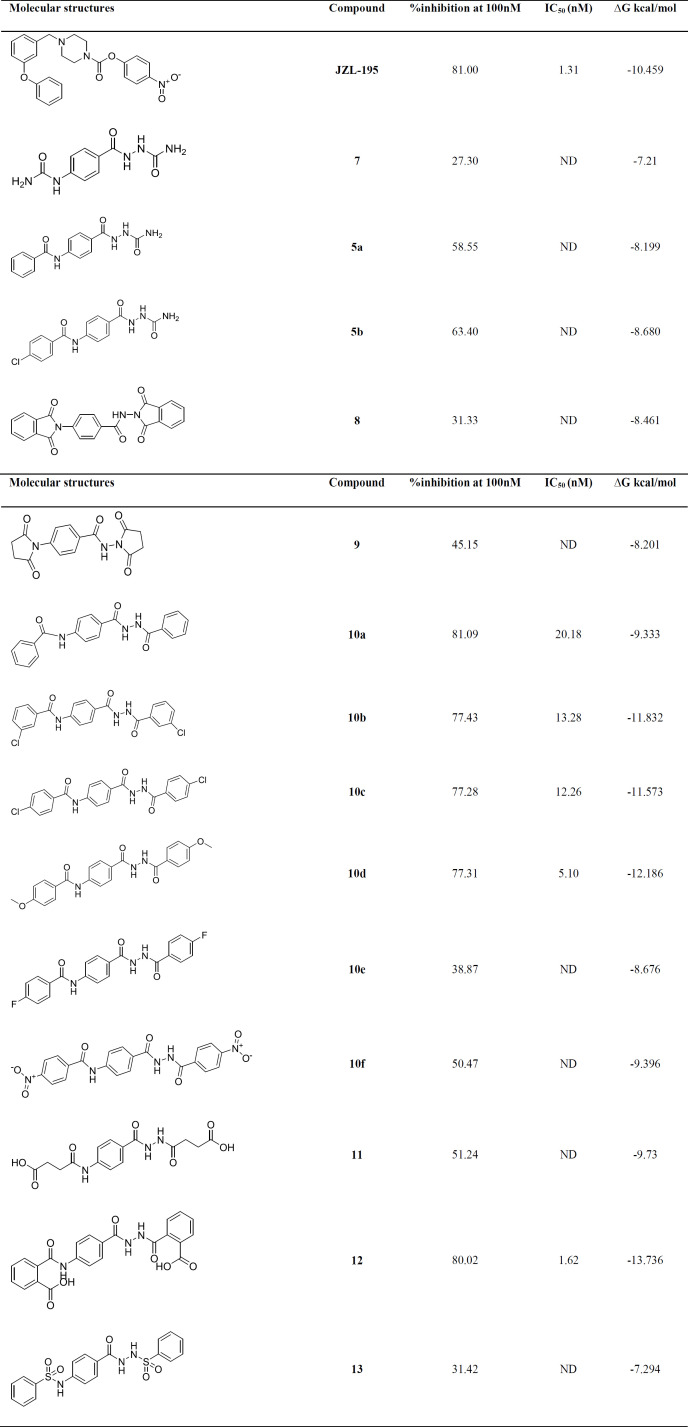

ND: not determined

## Conclusion

Novel 4-aminobenzohydrazide derivatives as potent FAAH inhibitors were synthesized and *in-vitro *evaluated. Compounds with a lipophilic phenolic group showed better inhibition effect and the presence of a carboxylic acid group on the phenol ring significantly increases the potency. According to the molecular docking studies, this effect is likely to be interpreted by the ionic interaction with Lys-142 in the active site. Compound **12** was the most potent compound with IC_50_ of 1.62 nM and comparable to JZL-195. These results seem to be valuable to design and develop of novel FAAH inhibitors.
